# Efficacy and safety of curcumin in psoriasis: preclinical and clinical evidence and possible mechanisms

**DOI:** 10.3389/fphar.2022.903160

**Published:** 2022-08-29

**Authors:** Shuo Zhang, Jiao Wang, Liu Liu, Xiaoying Sun, Yaqiong Zhou, Siting Chen, Yi Lu, Xiaoce Cai, Manqi Hu, Ge Yan, Xiao Miao, Xin Li

**Affiliations:** ^1^ Department of Dermatology, Yueyang Hospital of Integrated Traditional Chinese and Western Medicine, Shanghai University of Traditional Chinese Medicine, Shanghai, China; ^2^ Shanghai University of Traditional Chinese Medicine, Shanghai, China; ^3^ Institute of Dermatology, Shanghai Academy of Traditional Chinese Medicine, Shanghai, China

**Keywords:** curcumin (CUR), traditional Chinese medicine (TCM), Systematic review, Psoriasis, clincal, preclincal

## Abstract

**Background:** Psoriasis is a chronic and immune-mediated inflammatory skin disease. Many studies have shown that curcumin (CUR) has strong anti-inflammatory effects and can improve psoriasis; however, its efficacy and safety have not been confirmed, and the specific mechanism remains to be elucidated.

**Objective:** To evaluate the efficacy, safety, and possible mechanisms of CUR in the treatment of psoriasis.

**Methods:** The Cochrane Library, Embase, PubMed, Web of Science, China National Knowledge Infrastructure, Wanfang, and VIP (China Science and Technology Journal Database) were systematically searched for clinical trials and preclinical studies on the use of CUR in psoriasis treatment. All databases were searched from inception to January 2022. The meta-analysis was performed using RevMan 5.3 software.

**Results:** Our meta-analysis included 26 studies, comprising seven clinical randomized controlled trials and 19 preclinical studies. A meta-analysis of clinical trials showed that both CUR monotherapy and combination therapy improved Psoriasis Area and Severity Index (PASI) scores in patients compared to controls (standard mean difference [*std.MD*]: *−0.83%; 95%* confidence interval [*CI*]: −1.53 to 0.14; p = 0.02). In preclinical studies, CUR showed better performance in improving the phenotype of psoriatic dermatitis mice compared to controls, including total PASI score (std.MD: 6.50%; 95% CI: 10.10 to *−*2.90; p = 0.0004); ear thickness (*p = 0.01*); and the expression of inflammatory cytokines such as interleukin (IL)-17, tumor necrosis factor (TNF)-α, IL-17F, and IL-22 (*p < 0.05*). In cell studies, CUR inhibited cell proliferation (*p = 0.04*) and the cell cycle (*p = 0.03*) and downregulated the inflammatory cytokines IL-6 and IL-8 (*p < 0.05*).

**Conclusions:** CUR has excellent efficacy and broad potential to treat psoriasis in multiple ways. Its use also plays a crucial role in improving the psoriasis phenotype and reducing the inflammatory microenvironment. In conclusion, our findings suggest that CUR alone or in combination with other conventional treatments can effectively treat psoriasis.

## 1 Introduction

Psoriasis is an immune-mediated chronic inflammatory skin disease that affects 2%–3% of all individuals worldwide (Kopp et al., 2015). It typically presents as well-circumscribed, erythematous, and itchy plaques covered with silvery scales that can coalesce and cover large skin areas. Common sites affected by psoriasis include the trunk, extensor surfaces of the extremities, and scalp (Boehncke and Schön, 2015). The pathogenesis of psoriasis involves many factors, including immune abnormalities (Lowes et al., 2014), inflammatory activation (Rendon and Schäkel, 2019), cell proliferation and apoptosis (Shi et al., 2019), and neural mediators (Harvima and Nilsson, 2012). But its pathogenesis remains to be fully elucidated.

Psoriasis is characterized by epidermal keratinocyte (KC) hyperproliferation, abnormal differentiation and dermal inflammatory cell infiltration. IL-23/IL-17 axis is a key link in the pathogenesis of psoriasis. Triggering factors act on keratinocytes, causing activation of macrophage and dendritic cells that stimulate T_H_17 to produce pro-inflammatory cytokines, exacerbating the inflammatory response in psoriasis (Rivas Bejarano and Valdecantos, 2013; Fan et al., 2015; Griffiths et al., 2021). The current treatments for psoriasis include immunosuppressants (cyclosporine A and tacrolimus), vitamin D analogs, topical corticosteroids, retinoids, oral methotrexate, and cyclosporine, all of which have limitations in terms of treatment response and adverse effects (Deng et al., 2016). Biological drugs include secukinumab, which targets interleukin (IL)-17A; ustekinumab, which targets IL-12/IL-23; and infliximab, which antagonizes tumor necrosis factor (TNF)-α. Biologics are costly and may lose potency with prolonged use (Egeberg et al., 2018; Armstrong and Read, 2020). Therefore, complementary and alternative treatments for psoriasis need to be studied.

Curcumin (CUR) is the main active ingredient extracted from turmeric, a plant known for its medicinal properties and various pharmacological activities (Kocaadam and Şanlier, 2017; Chopra et al., 2021). Various pharmacological studies have shown that CUR has anti-inflammatory, antioxidant, anti-tumor, and anti-vascular remodeling effects (Aggarwal and Harikumar, 2009; Walker and Mittal, 2020; Mahjoob and Stochaj, 2021). Existing evidence suggests that CUR has therapeutic potential for a variety of human diseases (Oskouie et al., 2019; Kao et al., 2020; Mirzaei et al., 2021). It regulates various cell signaling molecules, including phosphorylase kinase; transferrin receptor; total cholesterol; transforming growth factor-β; pro-inflammatory cytokines (e.g., TNF-α, IL-17, IL-1β, and IL-6); STAT3; endothelin-1 apoptosis protein; nuclear factor-κB (NF-κB); cyclooxygenase-2; and antioxidants (Gupta et al., 2013; Nosrati-Oskouie et al., 2021; Pourbagher-Shahri et al., 2021). Preclinical studies have provided a solid basis for evaluating the bioavailability and safety of CUR in clinical trials (Vollono et al., 2019). A number of clinical trials have verified that CUR is safe and effective in the treatment of psoriasis (Rahmayunita et al., 2018; Mata et al., 2021; Marton et al., 2022), CUR has low toxicity but poor bioavailability, which may benefit patients with psoriasis as adjunctive therapy (Elmets et al., 2021).

However, the efficacy and mechanism of CUR in the treatment of psoriasis have not been fully elucidated. Therefore, we aimed to systematically review all published reports related to preclinical studies and clinical trials on the use of CUR in psoriasis treatment, as well as to quantitatively analyze its therapeutic effects and possible therapeutic targets from a mechanistic perspective.

## 2 Materials and Methods

### 2.1 Data sources and searches

The following electronic databases were searched: Cochrane Library, Embase, PubMed, Web of Science, China National Knowledge Infrastructure, Wanfang, and VIP (China Science and Technology Journal Database). Studies published in English and Chinese were searched in the databases. All databases were searched from inception to January 2022. The following MeSH (Medical Subject Headings) terms were used as search keywords to find studies that examined the effects of turmeric (Curcuma longa) or CUR on psoriasis: “curcumin,” “curcuma,” “turmeric,” “*Curcuma domestica*,” “*Curcuma longa*,” “psoriasis,” and “psoriases.”

### 2.2 Article evaluation and selection

Two independent reviewers (SZ and JW) screened the articles. In the first screening, related articles were identified from the titles and abstracts, and relevant articles were retrieved in full text and validated for inclusion in the systematic review. A third reviewer (LL) independently validated the selected articles.

### 2.3 Eligibility criteria for studies included in this review

**FIGURE 1:**
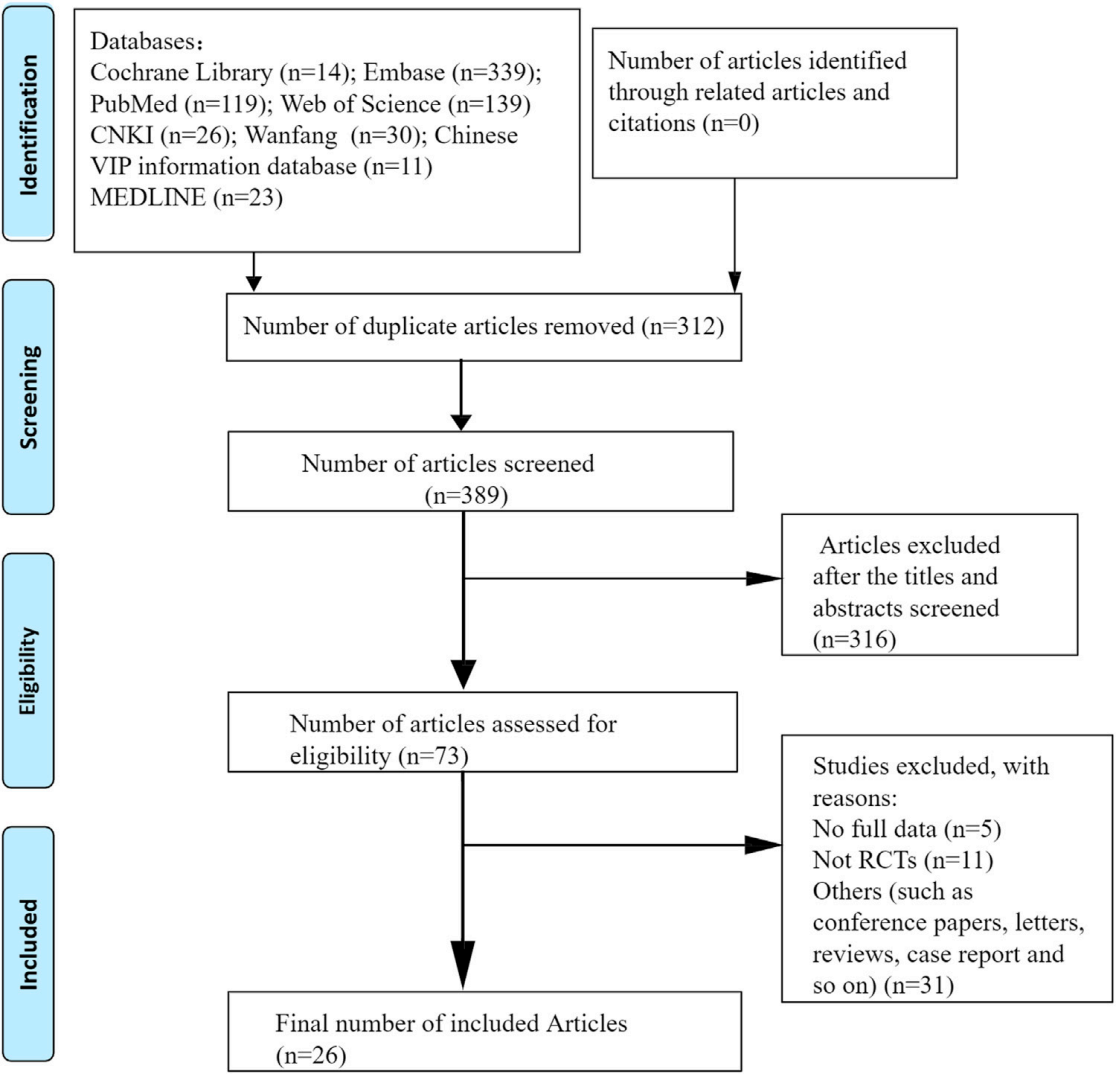
Flow diagram according to PRISMA (Preferred Reporting Items for Systematic Reviews and Meta-analyses) 2009.

All eligible clinical and preclinical studies were included in this systematic review. Randomized controlled trials that used CUR or CUR-related preparations, CUR-treated psoriasis-like mouse models with control comparisons, or psoriasis-like cell models with CUR interventions and control comparisons were included in this review. The exclusion criteria were articles lacking primary data, review articles, and studies published only in abstract form. Duplicate studies, cohort studies, meta-analyses, and conference abstracts were also excluded. A flowchart of the article selection process is shown in Figure 1.

### 2.4 Data collection and quality assessment

Basic information about the study was extracted from each article, comprising article title, year of publication, study design, sample size, mean or median age of the participants, sex, intervention of the experimental group and the control group, and study outcome. The Cochrane Collaboration tool for assessing risk of bias was used for the assessment of the risk of bias in clinical trials. The SYRCLE (Systematic Review Centre for Laboratory Animal Experimentation) risk-of-bias tool, which is based on the Cochrane Collaboration risk-of-bias tool, was used to judge the quality of animal studies (low, high, or unclear risk of bias) (Hooijmans et al., 2014; Liu et al., 2021).

### 2.5 Statistical methods

Statistical analysis was performed using RevMan (version 5.3; The Nordic Cochrane Centre, The Cochrane Collaboration, 2014, Copenhagen, Denmark). Standard mean differences (std.MDs) with 95% confidence intervals (CIs) were calculated for continuous variables, and odds ratios (ORs) with 95% CIs were calculated for binary variables. Heterogeneity was assessed using I^2^ statistics. To analyze the possible reasons for heterogeneity, we conducted a subgroup analysis. When I^2^>50%, we used the random-effects model to evaluate the overall effect; otherwise, the fixed-effects model was used (Qu, 2018).

## 3 Results

### 3.1 Literature search and study characteristics

#### 3.1.1 Literature search

A total of 398 relevant articles were identified by searching for subject headings and free words. After the removal of duplicates, 312 articles remained. 316 irrelevant articles were excluded by reading titles and abstracts. After the full-text assessment, 47 articles were excluded because they were not randomized controlled trials, did not include an appropriate comparator, or lacked usable data. Finally, 26 studies (seven clinical studies and 19 preclinical studies) were included in our systematic review and meta-analysis.

#### 3.1.2 Study characteristics

Among the seven clinical studies (Heng et al., 2000; Zhang et al., 2005; Antiga et al., 2015; Bnys et al., 2015; Carrion-Gutierrez et al., 2015; Bahraini et al., 2018; Bilia et al., 2018), two studies (Heng et al., 2000; Bnys et al., 2015) used topical treatment as an intervention, including topical CUR (alcoholic gel preparation containing 1% CUR) and topical starch-fortified turmeric baths. The other five studies used oral CUR, comprising curcuma extract, curcuma decoction, turmeric tonic, Meriva (a novel bioavailable lecithin-based delivery form of CUR), and nanocurcumin. The sample size of the seven trials ranged from one to 63. Four studies (Heng et al., 2000; Zhang et al., 2005; Bnys et al., 2015; Bahraini et al., 2018) used CUR as a monotherapy. Two studies (Bnys et al., 2015; Bahraini et al., 2018) compared CUR to placebo. Two studies (Heng et al., 2000; Zhang et al., 2005) compared CUR and positive controls (topical calcipotriol and indigo pills). Two studies (Antiga et al., 2015; Bilia et al., 2018) used CUR combination therapy and compared CUR plus active intervention and active intervention alone (acitretin and steroids). Six studies (Zhang et al., 2005; Antiga et al., 2015; Bnys et al., 2015; Carrion-Gutierrez et al., 2015; Bahraini et al., 2018; Bilia et al., 2018) used the Psoriasis Area Severity Index (PASI) score as an outcome measure, and three studies (Antiga et al., 2015; Bahraini et al., 2018; Bilia et al., 2018) reported adverse effects. The specific study characteristics are summarized in Supplementary Table S1.

Among the preclinical studies, eight were *in vivo* studies (Jain et al., 2016; Jia and Fang, 2017; Mao et al., 2017; Sun et al., 2017; Qu, 2018; Zhang et al., 2019; Jin et al., 2020; Badanthadka et al., 2021), and the treatment of imiquimod (IMQ)-induced psoriasis-like mice comprised oral (Jia and Fang, 2017; Qu, 2018) and topical (Jain et al., 2016; Mao et al., 2017; Sun et al., 2017; Zhang et al., 2019; Jin et al., 2020; Badanthadka et al., 2021) CUR. Four studies (Jain et al., 2016; Jia and Fang, 2017; Mao et al., 2017; Qu, 2018) used BALB/c mice, and four studies (Sun et al., 2017; Zhang et al., 2019; Jin et al., 2020; Badanthadka et al., 2021) used C57BL mice. Two studies (Jia and Fang, 2017; Qu, 2018) reported intervention with CUR gavage, and six studies (Jain et al., 2016; Mao et al., 2017; Sun et al., 2017; Zhang et al., 2019; Jin et al., 2020; Badanthadka et al., 2021) used a topical intervention method. Topical CUR has been formulated into different dosage forms with improved penetration and efficacy, including tacrolimus and CUR co-loading (Jain et al., 2016), CUR-loaded nanoparticles (Sun et al., 2017), CUR-loaded nanoparticles incorporated in silk fibroin hydrogel (Mao et al., 2017), and CUR-loaded hyaluronic acid-modified ethosomes (Zhang et al., 2019).

Eleven *in vitro* studies (Cho et al., 2007; Sun et al., 2012; Song et al., 2014; Esposito et al., 2015; Hung et al., 2015; Zhao et al., 2015; Varma et al., 2017; Wang WQ. et al., 2019; Wang ZJ. et al., 2019; Zhang et al., 2020; Yuyun et al., 2021) used HaCaT cells, which were treated with different concentrations of CUR or CUR combined with different preparations. Psoriasis related mechanisms in keratinocytes were induced using vascular endothelial growth factor (Wang ZJ. et al., 2019; Zhang et al., 2020), IL-22 (Zhao et al., 2015), IMQ (Varma et al., 2017), TNF-α (Cho et al., 2007; Sun et al., 2012; Yuyun et al., 2021), nanostructured lipid dispersions (Esposito et al., 2015), and pSG5.HA.mZac1 (Hung et al., 2015). Supplementary Tables S2, S3 summarize the characteristics of the included preclinical studies.

#### 3.1.3 Risk-of-bias Assessment of the Included Articles

RevMan 5.3 software was used to evaluate the risk of bias in the included clinical trials. Five articles (Antiga et al., 2015; Bnys et al., 2015; Carrion-Gutierrez et al., 2015; Bahraini et al., 2018; Bilia et al., 2018) mentioned randomization. Four studies (Antiga et al., 2015; Carrion-Gutierrez et al., 2015; Bahraini et al., 2018; Bilia et al., 2018) used double-blind methods. None of the studies described any other bias. The results of the risk of deviation are shown in a publication bias table (Supplementary Figures S1, S2). The SYRCLE risk-of-bias tool was used to evaluate the risk of bias in animal studies. None of the studies described allocation concealment or blinding, suggesting a high risk of bias. Moreover, baseline data were not provided, making it difficult to assess baseline characteristics. However, the experimental results were reported in detail. The other risks of bias were also low (Supplementary Figure S3).

### 3.2 Clinical trials

#### 3.2.1 Psoriasis area and severity index of CUR alone and CUR combination therapy for psoriasis

The PASI score, a gold standard indicator of psoriasis severity, was the primary outcome measure in the included clinical studies. Calculation of the PASI score mainly involves the evaluation of erythema, infiltration, and scaling. A meta-analysis of four studies (Bnys et al., 2015; Carrion-Gutierrez et al., 2015; Bahraini et al., 2018; Bilia et al., 2018) that evaluated the PASI score in patients treated with CUR alone or CUR combination therapy for psoriasis showed that CUR alone resulted in a statistically significant improvement in the PASI score compared to placebo (std.MD: −1.26%; 95% CI: −2.39 to −0.12; *p* = 0.03). When CUR was used alone, the result was not significantly different from that of the positive control; that is, the effect of CUR alone was not better than that of conventional treatment (std.MD: 0.22%; 95% CI: −0.29 to 0.72; *p* = 0.40). However, the combined effects of CUR and conventional therapy improved the PASI scores in patients compared with conventional therapy alone (std.MD: −0.91%; 95% CI: −1.34 to −0.48; *p* < 0.0001). The details are listed in Table 1.

**TABLE 1:** Subgroup analysis of PASI in clinical studies.

**Study**	**Comparison**	**Mean ± SD**		**std. Mean difference [95% CI]**	***p* value**
		**E**	**C**		
**1. Cur Alone**					
**1.1 Placebo Therapy**					
Bahraini et al. (2018)	Tur VS placebo	0.425 ± 0.125	0.52 ± 0.15	−0.67 [−1.31, −0.04]	
Shathirapathiy et al. (2015)	SFTBs VS Naturopathy	9.27 ± 5.47	22.83 ± 8.78	−1.83 [−2.44, −1.22]	
Subtotal (95% CI) I²=85%				−1.26 [−2.39,−0.12]	*p* = 0.03
**1.2 Positive Therapy**					
Zhang et al. (2005)	Cur VS IP	3.85 ± 2.70	3.33 ± 2.00	0.22 [−0.29, 0.72]	
Subtotal (95% CI)				0.22 [−0.29, 0.72]	*p* = 0.40
**2. Cur Combinated**					
Bilia et al. (2018)	Cur+Acitretin VS Acitretin	1.80 ± 1.04	3.95 ± 2.28	−1.18 [−1.96, −0.40]	
Antiga et al. (2015)	Meriva+Steroids VS Steroids	1.30 ± 1.11	2.40 ± 1.60	−0.79 [−1.30, −0.27]	
Subtotal (95% CI) I² = 0%				−0.91 [−1.34, −0.48]	*p* < 0.0001
Total (95% CI) Random-effects I² = 85%				−0.83 [−1.53, 0.14]	*p* = 0.02

Abbrevations: SFTBs, starch-fortified turmeric baths; Tur, turmeric; IP, Indigo pill; Cur, curcumin; E, experimental group; C, control group.

#### 3.2.2 PASI50, PASI75, and PASI90 of CUR for psoriasis

PASI50 refers to a 50% reduction in PASI. According to the Disease of Traditional Chinese Medicine Syndrome Diagnosis Curative Standard, a treatment that reduces skin lesions by ≥50% is considered effective. A PASI score of >50 is considered an indicator of the effectiveness of psoriasis treatment. A meta-analysis of two studies (Antiga et al., 2015; Bilia et al., 2018) described in Table 2 showed that CUR in combination with an active control drug was more effective than the active control drug alone in improving PASI50 (OR: 3.94%; 95% CI: 1.56–9.92; p = 0.004) and PASI75 in the 12th week (OR: 4.31%; 95% CI: 1.49–12.43; *p* = 0.007); however, no difference was observed for PASI90 in the 12th week (OR: 4.16%; 95% CI: 1.01–17.08; *p* = 0.05).

**TABLE 2:** Subgroup analysis of PASI 50, PASI 75, PASI 90 in clinical studies.

**Study**	**Comparison**	**E**		**C**		**Risk ratio [95% CI]**	***p* value**
		**No. of Events**	**Total**	**No. of Events**	**Total**		
**2.1 PASI 50**							
Bilia et al. (2018)	Cur+Acitretin VS Acitretin	13	15	10	15	3.25 [0.52, 20.37]	
Antiga et al. (2015)	Cur+Steroids VS Steroids	23	31	13	32	4.20 [1.44, 12.25]	
Total (95% CI) Random-effects I² = 0%						3.94 [1.56, 9.92]	*p* = 0.004
**2.2 PASI 75**							
Bilia et al. (2018)	Cur+Acitretin VS Acitretin	6	15	3	15	2.67 [0.52, 13.66]	
Antiga et al. (2015)	Cur+Steroids VS Steroids	12	31	3	32	6.11 [1.52, 24.54]	
Total (95% CI) Random-effects I² = 0%						4.31 [1.49, 12.43]	*p* = 0.007
**2.3 PASI 90**							
Bilia et al. (2018)	Cur+Acitretin VS Acitretin	5	15	2	15	3.25 [0.52, 20.37]	
Antiga et al. (2015)	Cur+Steroids VS Steroids	5	31	1	32	5.96 [0.65, 54.31]	
Total (95% CI) Random-effects I² = 0%						4.16 [1.01, 17.08]	*p* = 0.05

Abbrevations: Cur, curcumin; PASI, psoriasis area and severity Index; CI, confidence interval; E, experimental group; C, control group.

#### 3.2.3 Adverse events

Three studies reported adverse events. One of the studies (Antiga et al., 2015) reported diarrhea in one patient in the experimental group, but nausea in one patient and another one complained of a papular eruption on the face occurring in the control group. The other study (Bilia et al., 2018) reported nausea and vomiting in one patient, peeling of the palms in one patient, and mild cheilitis in six patients in the experimental group. One study (Bahraini et al., 2018) reported dry skin lesions in two individuals in the control group. The other included articles did not report any adverse reactions.

### *3.3 In Vivo* Preclinical trials

#### 3.3.1 Psoriasis area and severity index of CUR for psoriasis

Five studies (Jia and Fang, 2017; Sun et al., 2017; Zhang et al., 2019; Jin et al., 2020; Badanthadka et al., 2021) assessed the total scores in CUR-treated psoriasis model mice on day 6, and a meta-analysis showed that CUR significantly reduced the total scores compared to controls (std.MD: −6.50%; 95% CI: −10.10 to −2.90; *p* = 0.0004). Six studies (Jain et al., 2016; Mao et al., 2017; Sun et al., 2017; Zhang et al., 2019; Jin et al., 2020; Badanthadka et al., 2021) assessed the scores for erythema and scaling on day 6, and four studies (Jain et al., 2016; Mao et al., 2017; Jin et al., 2020; Badanthadka et al., 2021) assessed the scores for lesion thickness. A meta-analysis showed that CUR significantly improved erythema (std.MD: −2.88%; 95% CI: −4.57 to −1.19; p = 0.0008), scaling (std.MD: −3.19%; 95% CI: −5.17 to −1.21; *p* = 0.002), and lesion thickness (std.MD: 2.42−; 95% CI: 3.30 to −1.53; *p* < 0.00001) in psoriatic-like mice compared to controls. Overall, CUR significantly improved the PASI scores in IMQ-induced psoriasis-like mice. The details are listed in Table 3.

**TABLE 3:** Analysis of PASI scores in preclinical studies in vivo.

	Mean ± SD		Std.Mean difference [95%CI]	*p* value
Study						
	E	C		
**3.1 Total PASI Score**				*p* = 0.0004
Badanthadka et al., 2012	2.31 ± 0.97	6.03 ± 3.28	−1.42 [−2.75, −0.09]	
Jia, et al., 2017	0.56 ± 1.10	8.25 ± 1.53	−5.63 [−7.25, −4.00]	
Nan, et al., 2020	0.34 ± 0.12	8.69 ± 0.66	−16.25 [−24.26, −8.24]	
Sun et al., 2017	4.14 ± 1.25	9.60 ± 0.27	−5.57 [−8.52, −2.63]	
Zhang et al., 2019	8.02 ± 0.20	11.30 ± 0.15	−16.13 [−27.32, −4.95]	
Total (95% CI) Random-effects I^2^ = 88%			−6.50 [−10.10, −2.90]	
**3.2 Erythema**				*p* = 0.0008
Jain et al., 2016	0.49 ± 0.18	1.99 ± 0.85	−2.21 [−3.96, −0.45]	
Badanthadka et al., 2012	0.81 ± 0.30	1.62 ± 0.78	−1.27 [−2.55, 0.02]	
Mao et al., 2017	0.99 ± 0.39	3.95 ± 0.40	−7.23 [−9.61, −4.86]	
Nan, et al., 2020	4.14 ± 1.25	9.60 ± 0.27	−5.57 [−8.52, −2.63	
Sun et al., 2017	2.89 ± 0.17	3.11 ± 0.24	−1.03 [−2.27, 0.21]	
Zhang et al., 2019	2.65 ± 0.70	3.60 ± 0.24	−1.58 [−3.34, 0.18]	
Total (95% CI) Random-effects I^2^ = 82%			−2.88 [−4.57, −1.19]	
**3.3 Scaling**				*p* = 0.002
Jain et al., 2016	0.81 ± 1.12	2.61 ± 1.07	−1.48 [−2.98, 0.01]	
Badanthadka et al., 2012	1.35 ± 0.03	2.61 ± 1.17	−1.41 [−2.73, −0.08]	
Mao et al., 2017	1.22 ± 0.20	4.01 ± 0.41	−8.35 [−11.06, −5.65]	
Nan et al., 2020	0.18 ± 0.10	3.55 ± 0.52	−8.31 [−12.52, −4.10]	
Sun et al., 2017	3.11 ± 0.22	3.29 ± 0.29	−0.65 [−1.82, 0.53]	
Zhang et al., 2019	2.82 ± 0.52	3.78 ± 0.15	−2.18 [−4.22, −0.14]	
Total (95% CI) Random-effects I^2^ = 86%			−3.19 [−5.17, −1.21]	
**3.4 Thickness**				*P* <0.00001
Jain et al., 2016	0.79 ± 0.91	2.26 ± 1.10	−1.34 [−2.79, 0.11]	
Badanthadka et al., 2012	0.36 ± 0.33	1.81 ± 1.40	−1.32 [−2.62, −0.01]	
Mao et al., 2017	1.21 ± 0.21	4.00 ± 0.42	−8.11 [−10.75, −5.48]	
Nan, et al., 2020	0.15 ± 0.06	3.58 ± 0.57	−7.81 [−11.79, −3.83]	
Total (95% CI) Random-effects I^2^ = 90%			−2.42 [−3.30, −1.53]	

Abbrevations: PASI, psoriasis area severity Index; E, experimental group; C, control group.

#### 3.3.2 Ear thickness

A meta-analysis of four studies (Jain et al., 2016; Sun et al., 2017; Zhang et al., 2019; Badanthadka et al., 2021) in Table 4 showed that psoriasis-like mice had significantly reduced ear thickness after 6 days of CUR treatment compared to controls (std.MD: 1.80%; 95% CI: −3.20 to −0.41; *p* = 0.01).

**TABLE 4:** Analysis of ear thickness in preclinical studies in vivo.

	Mean ± SD		std.Mean difference [95%CI]	*p* value
Study						
	E	C		
**4.1 epidermal Thickness**				*p* = 0.01
Jain, et al., 2016	0.28 ± 0.03	0.34 ± 0.08	−0.90 [−2.24, −0.44]	
Badanthadka, et al., 2021	0.23 ± 0.02	0.25 ± 0.01	−1.17 [−2.43, −0.10]	
Sun, et al., 2017	0.18 ± 0.17	0.95 ± 0.06	−5.58 [−8.52, −2.63]	
Zhang, et al., 2019	2.67 ± 1.13	4.03 ± 0.02	−1.48 [−3.20, 0.24]	
Total (95% CI) Random-effects I^2^ = 64%			−1.80 [−3.20, −0.41]	

Abbrevations: E, experimental group; C, control group.

#### 3.3.3 Cytokines

Two studies (Jain et al., 2016; Jin et al., 2020) evaluated inflammatory factors such as IL-17 and TNF-α in psoriasis-like mice after CUR treatment, using enzyme-linked immunosorbent assay. As expected, CUR reduced the release of inflammatory cytokines compared to the control group (std.MD: −1.35%; 95% CI: −2.58 to −0.12; *p* = 0.03 for IL-17 and std.MD: −3.82%; 95% CI: −6.97 to −0.66; *p* = 0.02 for TNF-α). Two studies (Sun et al., 2017; Zhang et al., 2019) assessed IL-17F, IL-22, and TNF-α using quantitative real-time polymerase chain reaction. Consistent with the above results, CUR reduced the release of IL-17F (std.MD: 2.84%; 95% CI: −5.04 to −0.64; p = 0.01) and IL-22 (std.MD: −4.42%; 95% CI: −7.31 to −1.52; *p* = 0.003) compared to the control group. However, no effect on TNF-α was observed (std.MD: −5.53%; 95% CI: −21.23 to 10.17; *p* = 0.49). The details are listed in Table 5.

**TABLE 5:** Analysis of cytokines in preclinical studies in vivo.

	Mean ± SD		std.Mean difference [95%CI]	*p* value
Study						
	E	C		
**ELISA**				*p* = 0.03
** 5.1 IL-17**				
Jain, et al., 2016	0.20 ± 0.07	0.42 ± 0.01	−2.16 [−3.89, −0.42]	
Nan, et al., 2020	1.36 ± 0.15	1.63 ± 0.38	−0.86 [−2.07, 0.34]	
Total (95% CI) Random-effects I^2^ = 30%			−1.35 [−2.58, −0.12]	
** 5.2 TNF-α**				*p* = 0.02
Jain, et al., 2016	0.42 ± 0.33	1.04 ± 0.15	−2.18 [−3.93, −0.44]	
Nan, et al., 2020	20.86 ± 2.00	33.50 ± 2.53	−5.40 [−6.98, −3.83]	
Total (95% CI) Random-effects I^2^ = 86%			−3.82 [−6.97, −0.66]	
**RT-PCR**				
** 5.3 IL-17F**				*p* = 0.01
Sun, et al., 2017	0.57 ± 0.32	20.19 ± 6.29	−4.07 [−6.35,−1.78]	
Zhang, et al., 2019	13.59 ± 2.87	20.69 ± 3.87	−1.81 [−3.68, 0.05]	
Total (95% CI) Random-effects I^2^ = 55%			−2.84 [−5.04, −0.64]	
** 5.4 IL-22**				*p* = 0.003
Sun, et al., 2017	0.20 ± 0.25	4.52 ± 1.58	−3.53 [−5.59, −1.47]	
Zhang, et al., 2019	20.16 ± 6.32	98.49 ± 12.53	−6.86 [−11.78, −1.94]	
Total (95% CI) Random-effects I^2^ = 34%			−4.42 [−7.31, −1.52]	
** 5.5 TNF-α**				*p* = 0.49
Sun, et al., 2017	0.90 ± 0.40	0.14 ± 0.41	1.73 [0.32, 3.14]	
Zhang, et al., 2019	9.38 ± 2.77	86.48 ± 5.99	−14.37 [−24.35, −4.39]	
Total (95% CI) Random-effects I^2^ = 90%			−5.53 [−21.23, 10.17]	

Abbrevations: IL, interleukin; TNF, tumor necrosis factor; E, experimental group; C, control group.

### *3.4 In vitro* Preclinical trials

#### 3.4.1 Cell proliferation, apoptosis rate, and cell cycle

Three studies (Zhao et al., 2015; Wang ZJ. et al., 2019; Zhang et al., 2020) analyzed the effect of CUR on HaCaT cell proliferation in cell experiments; two studies (Sun et al., 2012; Wang WQ. et al., 2019) analyzed the apoptosis rate; and two studies (Hung et al., 2015; Wang WQ. et al., 2019) analyzed the cell cycle. The results of the meta-analysis in Table 6 showed that CUR intervention inhibited cell proliferation (std.MD: −3.88%; 95% CI: −7.58 to −0.17; *p* = 0.04) and the cell cycle (std.MD: −2.22%; 95% CI: −4.24, −0.21; *p* = 0.03) compared to the control group, and the difference was statistically significant. However, it had no effect on the apoptosis rate (std.MD: 6.44%; 95% CI: −8.45 to 21.34; *p* = 0.40).

**TABLE 6:** Analysis of cell proliferation in preclinical studies in vitro.

	Mean ± SD		Std.Mean difference [95%CI]	*p* value
Study						
	E	C		
**6.1 cell proliferation**				*p* = 0.04
Wang, et al., 2019 (1)	0.35 ± 0.05	0.42 ± 0.09	−0.84 [−2.34, 0.66]	
Zhang, et al., 2020	1.74 ± 0.13	4.05 ± 0.30	−9.22 [−13.87, −4.58]	
Zhao, et al., 2015	0.25 ± 0.03	0.43 ± 0.06	−3.43 [−5.72, −1.13]	
Total (95% CI) Random-effects I^2^ = 85%			−3.88 [−7.58, −0.17]	
**6.2 Apoptosis Rate**				*p* = 0.4
Sun, et al., 2012	12.80 ± 4.75	9.20 ± 5.31	0.65 [-0.65, 1.94]	
Wang, et al., 2019 (2)	31.35 ± 1.53	5.32 ± 0.94	16.40 [0.48, 32.32]	
Total (95% CI) Random-effects I^2^ = 73%			6.44 [-8.45, 21.34]	
**6.3. Cell Cycle**				*p* = 0.03
Hung, et al., 2015	24.20 ± 0.14	29.19 ± 1.37	−4.10 [−8.37, 0.17]	
Wang, et al., 2019 (2)	16.80 ± 2.12	22.30 ± 3.02	−1.69 [−3.97, 0.60]	
Total (95% CI) Random-effects I^2^ = 0%			−2.22 [−4.24, −0.21]	

Abbrevations: E, experimental group; C, control group.

#### 3.4.2 IL-6 and IL-8

The results of a meta-analysis of two studies (Sun et al., 2012; Yuyun et al., 2021) in Table 7 showed that CUR as an intervention significantly reduced the expression of inflammatory factors such as IL-6 (std.MD: 4.07%; 95% CI: −6.31 to −1.83; p = 0.0004) and IL-8 (std.MD: −4.19%; 95% CI: −8.11 to -0.27; *p* = 0.04) in a TNF-α-induced HaCaT cells.

**TABLE 7:** Analysis of inflammatory cytokines in preclinical studies in vitro

	Mean ± SD		Std.Mean difference> [95%CI]	*p* value
Study						
	Experiment	Control		
7.1 IL-6				*p* = 0.0004
Sun, et al., 2017	11.20 ± 3.75	27.50 ± 3.87	−3.86 [−6.37, −1.36]	
Yuyun, et al., 2021	15.60 ± 4.51	38.70 ± 2.8	-4.90 [−9.90, 0.09]	
Total (95% CI) Random-effects I^2^ = 0%			−4.07 [-6.31, −1.83]	
7.2 IL-8				*p* = 0.04
Sun, et al., 2017	58.00 ± 19.10	325.00 ± 49.60	−6.42 [−10.24, −2.60]	
Yuyun, et al., 2021	8.90 ± 3.75	20.80 ± 4.20	−2.39 [−5.20, 0.42]	
Total (95% CI) Random-effects I^2^ = 64%			−4.19 [−8.11, −0.27]	

AbbreviationsIL, interleukin; E, experimental group; C, control group.

## 4 Discussion

### 4.1 Summary of evidence

To our knowledge, this is the first systematic review of the clinical efficacy, safety, and potential mechanisms of CUR and its active ingredients in the treatment of psoriasis. We conclude that CUR has anti-inflammatory properties and improves psoriasis by inhibiting KC proliferation and the release of inflammatory factors. Thus, it acts as a key link in the immune inflammatory response in the pathogenesis of psoriasis. However, the conventional treatment for psoriasis is un satisfactory. First-line drugs, such as methotrexate, often cause side effects such as bone marrow suppression and hepatotoxicity. Numerous studies have confirmed the safety and efficacy of CUR for treating psoriasis. Our study once again confirmed that CUR can effectively alleviate psoriatic skin lesions alone or incombination with other drugs. Thus, CUR can be a complementary alternative therapy for psoriasis to reduce the side effects of its conventional treatments.

### 4.2 Limitations

Our study had some limitations. First, the sample size of the included clinical trials was small, the methodological quality of some of the included studies was not high, and there was high heterogeneity among some outcome indicators. Second, a doctoral thesis (not a standard peer-reviewed journal article) was included in the literature that we analyzed. Finally, most of the included studies investigated a single mechanism, which makes it difficult to identify the key targets of CUR in the treatment of psoriasis. Thus, further research is required to illustrate how and to what extent CUR or its derivatives can be used safely and efficiently as an adjuvant or main therapy for psoriasis.

### 4.3 Implications

*Curcuma longa L* is a natural herb, and CUR is the main active ingredient extracted from *Curcuma longa L*. CUR has been proposed as a treatment for various skin diseases, such as scleroderma, psoriasis, eczema, and skin cancer, by scientists and clinicians worldwide (Vaughn et al., 2016).

In clinical studies, we analyzed the efficacy of CUR according to the severity of skin lesions as assessed using the PASI score, PASI50, PASI75, and PASI90 in patients with psoriasis. CUR is considered a prominent anti-psoriatic compound owing to its potent antioxidant and anti-inflammatory properties (Wu et al., 2020). In one clinical study (Sarafian et al., 2015), 34 patients applied a turmeric microemulsion to plaques on the right arm and a control placebo to symmetrical plaques on the left arm. The results showed a statistically significant reduction in erythema, desquamation, and plaque thickness after CUR treatment. Kurd et al. (2008) conducted a phase II, open-label, Simon’s two-stage trial of 4.5 g/d oral CUR C3 complex in patients with plaque psoriasis. Oral CUR was well tolerated and safe in patients with psoriasis. All adverse events were mild and limited to gastrointestinal discomfort, heat intolerance, or hot flashes. Only two of the 12 participants achieved a PASI score of 75. This low response rate may be due to the low bioavailability of oral CUR.

Multiple human clinical trials have shown that CUR is safe and effective. The reported toxic side effects of oral CUR on the human body are minimal. Even at higher doses, there are no obvious toxic and side effects. However, CUR is less absorbed when taken orally, and there is a first-pass effect (Pan and Wang, 2012). The low bioavailability *in vivo* limits the promotion and use of CUR (Liu et al., 2016). To improve its efficacy and bioavailability, CUR dosage forms have been modified; for example, CUR formulated as nanoparticles showed higher solubility and favorable safety profile (Bilia et al., 2018). Meriva, a novel bioavailable lecithin-based delivery form of CUR, increased the plasma curcuminoid concentrations after its oral administration (Antiga et al., 2015). CUR esterified with mycophenolic acid showed enhanced oral bioavailability (Yuyun et al., 2021). As for the treatment of skin diseases, topical drugs can be directly applied to the affected area of the skin. CUR has been formulated into a variety of topical dosage forms and products to increase the effect of CUR transdermal absorption (Mohanty and Sahoo, 2017; Sun et al., 2017; Zhang et al., 2019; Jin et al., 2020). The use of skin-permeating nanoparticles (NPs) can facilitate delivery of CUR to the deeper layers of the skin (Mao et al., 2017). Topical CUR avoids the liver first-pass effect and can be directly applied to the affected area of the skin to improve the therapeutic effect of psoriasis.

Immune system abnormalities are an important mechanism in the pathogenesis of psoriasis; activated T cells and dendritic cells are critical in maintaining the psoriatic phenotype; the IL-23 and type 17 T cell axis is the central link in development; and keratinocyte (KC) changes are secondary to abnormal cellular immunity (Luo et al., 2021). Our research found that CUR affects many upstream and downstream links in the psoriatic inflammatory cascade.

**FIGURE 2:**
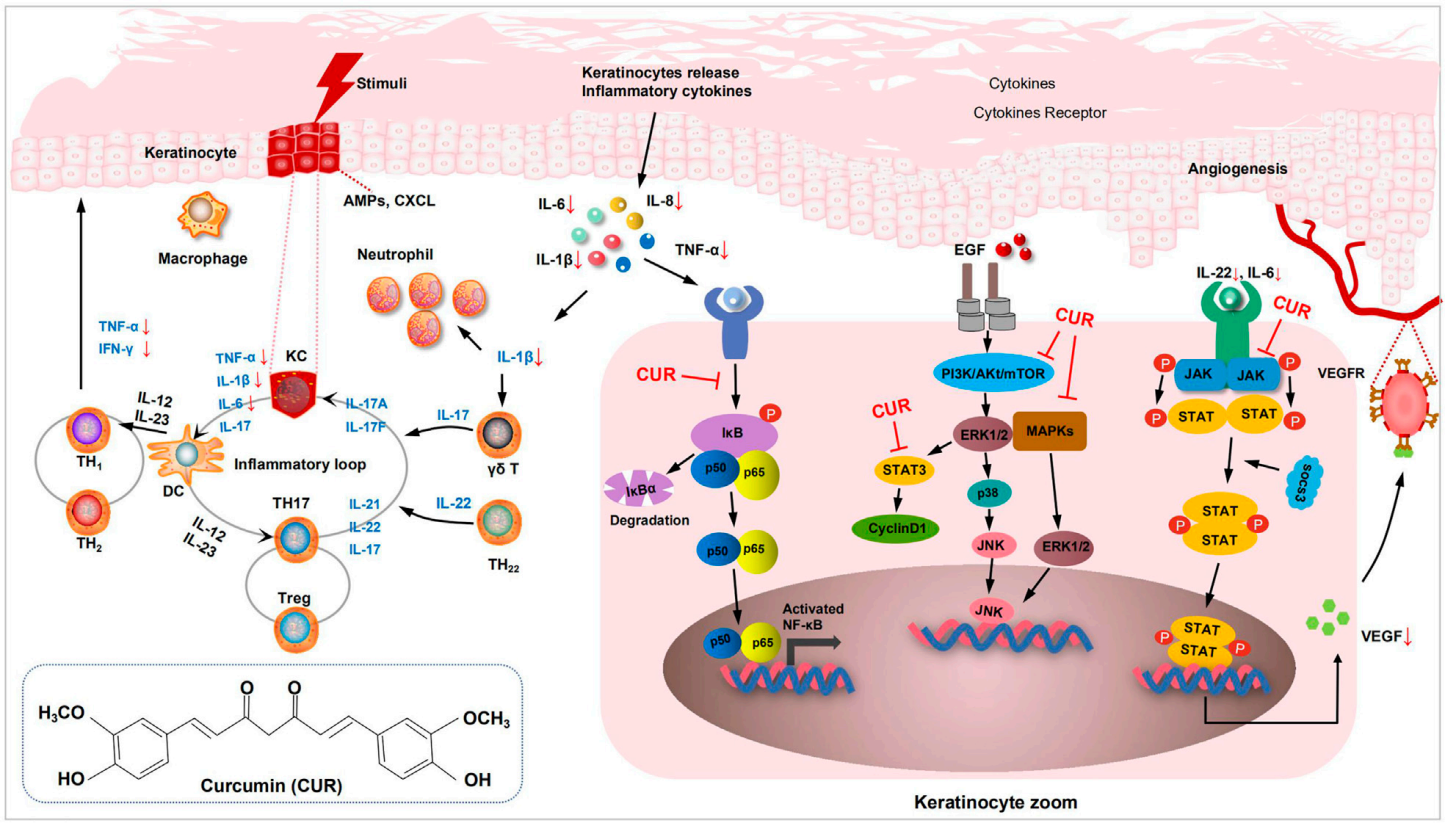
Diagram of the mechanism of curcumin (CUR) in in vitro preclinical studies. Mechanism of CUR in psoriatic dermatitis. The cytokines IL-12 and IL-23 released by DCs stimulate Th1 cells to produce TNF-α and INF-γ, and stimulate Th17 cells to produce IL-22 and other cytokines. IL-17 and TNF-α induced KCs to produce pro-inflammatory factors, such as IL-6 and IL-8, causing massive accumulation of neutrophils and activation of the NF-κB signaling pathway. IL-22 secreted by Th17 cells activates the JAK-STAT3 and MAPK signaling pathway. CUR inhibited IL-22 induced phosphorylation of STAT3, and reduces vascular proliferation by inhibiting VEGF. CUR reduced the secretion of inflammatory factors by inhibiting KCs and further blocks the activation of the NF-κB, JAK-STAT3 and MAPK signaling pathway. DCs, dendritic cells; KCs, keratinocytes; VEGF, vascular endothelial growth factor; Th17, T helper 17; IL, interleukin; NF-κB, nuclear factor-κB; JAK-STAT3, (Janus tyrosine Kinase)-(Signal Transducer and Activator of Transcription).

For preclinical studies, we performed a meta-analysis of relevant assays in animal and cellular models of psoriasis following the CUR intervention. CUR inhibits the expression of inflammatory cytokines (TNF-α and IL-6) and decreases the levels of the key inflammatory factor IL-17 A in the skin of psoriatic mice (Mao et al., 2017; Jin et al., 2020). CUR also exerts anti-inflammatory effects that improve psoriasis by inhibiting the signaling pathways of mitogen-activated protein kinase (MAPK) proteins P38, ERK, and JNK (Yuyun et al., 2021). Specifically, CUR inhibited the MAPK (P38, JNK, ERK) signaling pathways and downregulated the expression of IL-1β, IL-6, TNF-α, and other pro-inflammatory cytokines in psoriasis. Psoriasis activates several signaling pathways that increase cell proliferation, which can be mediated through the activation of the transcription factor NF-ĸB. NF-ĸB activation requires the removal of its inhibitory protein, IĸB, by phosphorylation of its kinase, which prevents NF-κB activation by inhibiting IĸBα phosphorylation and degradation (Liczbiński et al., 2020). Cho et al. (2007) found that CUR dramatically inhibited the TNF-α-induced activation of p65 NF-κB induced by TNF-α-treated HaCaT cells. As analyzed by *in vitro* studies, CUR inhibited HaCaT cell proliferation and exerted anti-inflammatory effects. Cyclin D1 is a positive regulator of the cell cycle, promoting cell cycle progression from the G0/G1 phase to the S phase, mitosis, and cell proliferation. CUR can arrest KCs in the G0/G1 phase and inhibit cyclin D1 in KCs and Bcl-2 protein expression. The therapeutic effect of CUR may be related to the downregulation of cyclin D1 and Bcl-2 expression, and the arrest of cells in the G0/G1 phase (Wang et al., 2019a). The mechanism of CUR in the treatment of psoriasis is shown in Figure 2.

In summary, we evaluated the efficacy and safety of CUR in the treatment of psoriasis through a meta-analysis of clinical studies and elucidated its specific mechanisms based on preclinical studies that used a psoriasis-like mouse model and a psoriasis cell model. Large-scale, high-quality, multi-center studies are needed to confirm our conclusion, so as to increase market development efforts and to provide more methods and strategies for the treatment of psoriasis.

## 5 Conclusion

CUR can improve psoriatic skin lesions effectively with few adverse effects. CUR exerts ameliorating effects on psoriasis by reducing the release of inflammatory factors, thus inhibiting cell proliferation and cell cycle through multiple signaling pathways. Therefore, the findings of this study support CUR as a promising complementary and alternative therapy for managing psoriasis.

## Data Availability

The original contributions presented in the study are included in the article/Supplementary Material, further inquiries can be directed to the corresponding authors.

## Glossary

CUR,: curcumin;
CNKI,: China Network Knowledge Infrastructure;
VIP,: China Science and Technology Journal Database;
MeSH,: Medical Subject Headings;
TNF-α,: tumor necrosis factor-α;
IFN-γ,: interferon-γ;
PASI,: Psoriasis Area and Severity Index;
IMQ,: imiquimod;
TCM,: traditional Chinese medicine;
AEs,: adverse events;
IL,: interleukin;
TGF-β,: transforming growth factor;
COX-2,: cyclooxygenase;
NF-κB,: nuclear factor-κB;
CI,: confidence interval;
SFTBs,: starch-fortified turmeric baths;
Tur,: turmeric;
IP,: indigo pill;
KC,: keratinocyte;
WB,: Western blot;
PCR,: quantitative real-time polymerase chain reaction;
ELISA,: enzyme-linked immunosorbent assay;
FCM,: flow cytometry;
iNV,: involucrin;
CCK-8,: Cell Counting Kit-8;
FACS,: fluorescence-activated cell sorting;
MAPK,: mitogen-activated protein kinase;
VEGF,: vascular endothelial growth factor;
MPA-CUR,: mycophenolic acid-esterified curcumin;
HE,: hematoxylin and eosin;
IHC,: immunohistochemistry;
TAC-CUR,: tacrolimus and curcumin co-loading;
TAC,: tacrolimus;
CUR-NPs-gel,: curcumin-loaded nanoparticles incorporated in silk fibroin hydrogel
