# Supplementary material for: Efficacy and safety of curcumin in psoriasis: preclinical and clinical evidence and possible mechanisms
Source: Front Pharmacol. 2022 Aug 29;13:903160. doi: 10.3389/fphar.2022.903160 (PMC9477188; doi:10.3389/fphar.2022.903160)
Supplement: Supplementary file 1 [file Table1.docx]

| **Table S1. Characteristics of the included clinical studies** | | | | | | | | | | | | | | |
| --- | --- | --- | --- | --- | --- | --- | --- | --- | --- | --- | --- | --- | --- | --- |
| **Author, year** | **Sample size** | | **Average age (years) (mean±SD)** | | **Sex (M/F)** | | **Duration of psoriasis (years) (mean±SD)** | | **Intervention** | | **Duration of  treatment (weeks)** | **Outcomes** | **Adverse  events** | |
|  | **E** | **C** | **E** | **C** | **E** | **C** | **E** | **C** | **E** | **C** |  |  | **E** | **C** |
| Heng, *et al* 2000 | 10 | 10 | 58.2±14.5 | 62.5±14.7 | N/A | N/A | 4 | 2 | Topical Cur (alcoholic gel preparation containing 1% Cur) | Topical calcipotriol ointment | N/A | PhK, TRR, CD8, parakeratosis | N/A | N/A |
| Zhang, *et al* 2005 | 30 | 30 | 36.3±9.6 | 38.1±7.8 | 17/13 | 20/10 | 9.80±1.80 | 11.00±2.30 | Oral Cur (decoction of curcuma) | Indigo pill | 8 | PASI, efficiency | N/A | N/A |
| Carrion-Gutierrez, *et al* 2015 | 11 | 10 | 44.4±11.6 | 37.9±12 | 9/2 | 4/6 | 6.50±4.10 | 5.40±3.70 | Oral Cur + VLRT | Oral Cur + VLST | N/A | PASI, PGA | N/A | N/A |
| Antiga, *et al* 2015 | 31 | 32 | 37 | 41 | 14/17 | 18/14 | N/A | N/A | Oral Cur (Meriva) + topical steroids | Oral placebo + topical steroids | 16 | PASI, Efficiency, AEs | 1 | 2 |
| Shathirapathiy, *et al* 2015 | 30 | 30 | 40.81±13.39 | 32.33±8.7 | 21/9 | 19/11 | N/A | N/A | Topical SFTBs | Naturopathy (massage, yoga, hydrotherapy, diet therapy) | 9 | PASI, BSA | N/A | N/A |
| Bahraini, *et al* 2018 | 20 | 20 | 29 | 44 | 6/9 | 3/12 | N/A | N/A | Oral Cur (turmeric tonic) | Oral placebo | 9 | PASI, DLQI, AEs | N/A | 2 |
| Bilia, *et al* 2018 | 15 | 15 | N/A | N/A | 7/8 | 9/6 | 5.40 | 4.80 | Oral Cur (nanocurcumin) + acitretin | Oral acitretin | 16 | PASI, efficiency, AEs | 8 | 9 |
| **Abbreviations:** Cur, curcumin; VLRT, real visible light phototherapy; VLST, simulated visible light phototherapy; PhK, phosphorylase kinase; TRR, keratinocyte transferrin receptor; SFTBs, starch-fortified turmeric baths; PGA, Physician Global Assessment; PASI, Psoriasis Area Severity Index; DLQI, Dermatology Life Quality Index; BSA, body surface area; AEs, adverse events; N/A, not applicable; E, Experimental group; C, Control group. | | | | | | | | | | | | | | |
