# Supplementary figures and images for: Efficacy and safety of curcumin in psoriasis: preclinical and clinical evidence and possible mechanisms
Source: Front Pharmacol. 2022 Aug 29;13:903160. doi: 10.3389/fphar.2022.903160 (PMC9477188; doi:10.3389/fphar.2022.903160)

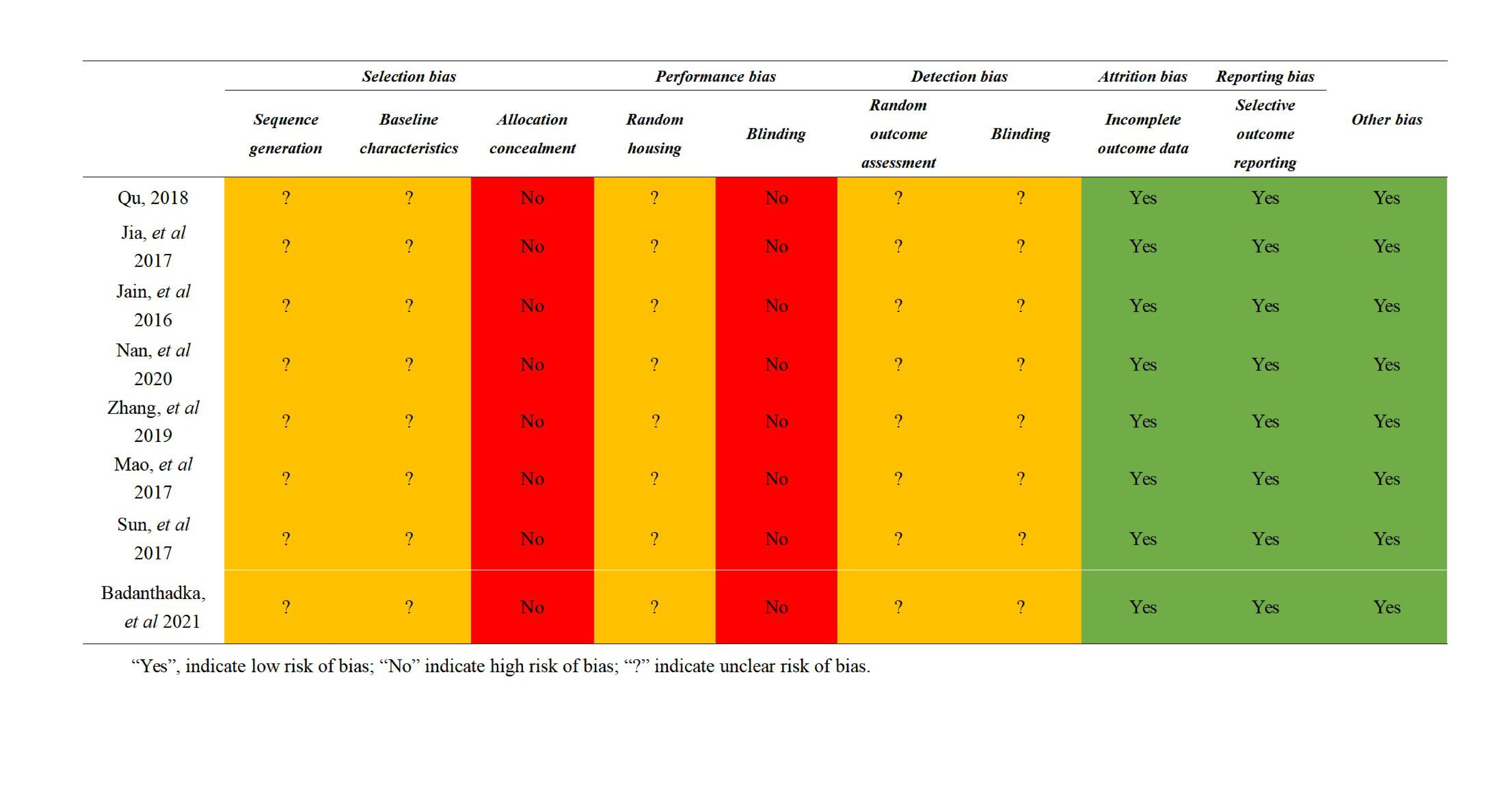

Supplement: Supplementary file 2 [file Image3.TIF]

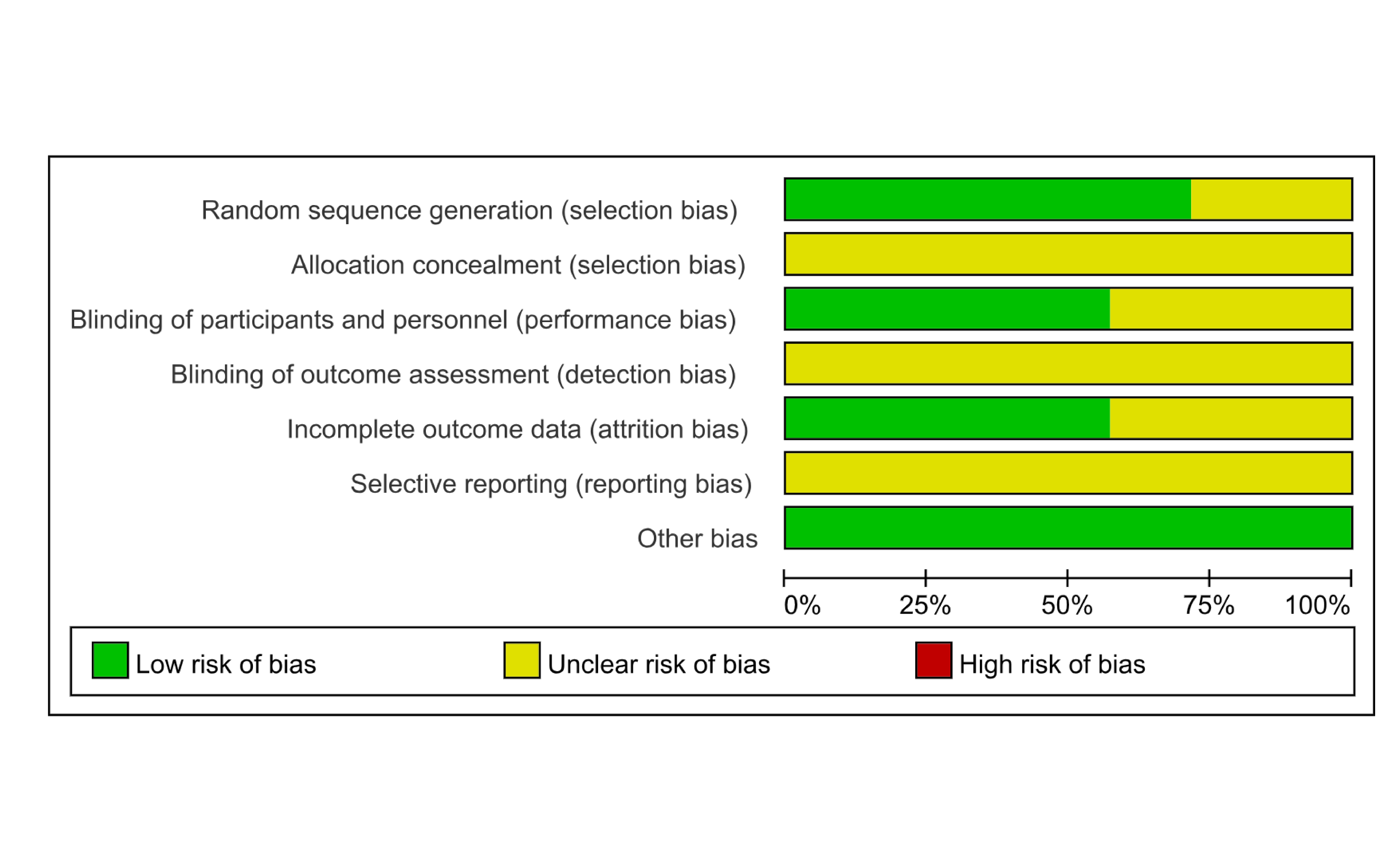

Supplement: Supplementary file 4 [file Image1.TIF]
