# Supplementary material for: Efficacy and safety of curcumin in psoriasis: preclinical and clinical evidence and possible mechanisms
Source: Front Pharmacol. 2022 Aug 29;13:903160. doi: 10.3389/fphar.2022.903160 (PMC9477188; doi:10.3389/fphar.2022.903160)
Supplement: Supplementary file 5 [file Table2.docx]

| **Table S2. Characteristics of the included *in vivo* preclinical studies** | | | | | | |
| --- | --- | --- | --- | --- | --- | --- |
| **Author, year** | **Species (sex, n: experimental/control group)** | **Weight (g)** | **Model (method)** | **Method of administration** | | **Outcome indicator** |
|  |  |  |  | **E** | **C** |  |
| Jain, *et al* 2016 | BALB/c mice (male, N/A) | N/A | Psoriasis model  (42 mg/d IMQ, 8 d) | 100 mg/d, TAC-CUR liposphere gel, g.t.; IMQ applied on the back, 42 mg/d, 6 d | CON group: Vaseline applied on the back, 42 mg/d, 6 d IMQ group: IMQ applied on the back, 42 mg/d, 6 d **TAC** group: TAC 100 mg/d; IMQ applied on the back, 42 mg/d, 6 d | 1. PASI 2. Histology staining 3. Epidermal thickness 4. IL-17 5. TNF-α 6. IL-22 7. IL-23 |
| Jia, *et al* 2017 | BALB/c mice (male/female, 16/16) | 19–21 | Psoriasis model  (42 mg/d 4% IMQ, 6 d) | 3 mL turmeric soup, qd, i.g.;  4% IMQ applied on the back, 42 mg/d, 14 d | CON group: 3 mL distilled water, qd, p.o.; cream applied on the back, 14 d IMQ group: 43 mL distilled water, qd, p.o.; 4% IMQ applied on the back, 42 mg/d, 14 d | 1. PASI 2. Epidermal thickness 3. Histology staining 4. IL-6 5. IL-8  6. TNF-α |
| Mao, *et al* 2017 | BALB/c mice (male, N/A) | 8–11 | Psoriasis model  (4 mg/d IMQ, 8 d) | CUR-NPs-gel, 500 µg, qid, five times, g.t.; IMQ applied on the back, 4 mg/d, 8 d | CON group: N/A **Clob** group: N/A; IMQ applied on the back, 4 mg/d, 8 d | 1. PASI 2. Epidermal thickness 3. Histology staining 2. IHC 3. TNF-α 4. IL-6 5. NF-κB |
| Sun, *et al* 2017 | C57BL/6 mice (female, N/A) | 7–9 | Psoriasis model (62.5 mg/d 5% IMQ, 10 d) | 50 nm Cur-NPs, 0.25 mg/d/mouse, g.t.;  5% IMQ applied on the back, 62.5 mg/d, 10 d | CON group: N/A  IMQ group: IMQ applied on the right ear, 25 mg/cm^2^/d, 10 d **TAC** group: 45 mg/cm^2^/d, IMQ applied on the right ear, 25 mg/cm^2^/d, 10 d | 1. PASI  2. Ear thickness  3. Spleen weight  4. IL-17F  5. IL-22  6. IL-6  7. IL-23  8. IL-6  9. IL-1β  10. TNF-α  11. IFN-γ  12. STAT3 |
| Qu, 2018 | BALB/c mice (female, 15/15) | 18–20 | Psoriasis model  (42 mg/d 4% IMQ, 6 d) | 400 mg/kg turmeric soup qd, i.g.; 4% IMQ applied on the back, 42 mg/d, 6 d | CON group**:** distilled water, qd, p.o.; Vaseline applied on the back, 6 d IMQ group: distilled water, qd, p.o.; 4% IMQ applied on the back, 42 mg/d, 6 d | 1. PASI 2. HE dye 3. ASC  4. NLRP3 |
| Zhang, *et al* 2019 | C57BL/6 mice (male, 18/12) | 8–11 | Psoriasis model (25 mg/cm^2^/d IMQ, 10 d) | HA-ES, 0.3 mL/cm^2^, g.t.;  IMQ applied on the right ear, 25 mg/cm^2^/d, 10 d | CON group: no treatment  IMQ group: IMQ applied on the right ear, 25 mg/cm^2^/d, 10 d **CP** group: 45 mg/cm^2^/d; IMQ applied on the right ear, 25 mg/cm^2^/d, 10 d | 1. PASI 2. Histology staining  3. Spleen index 4. IL-17A  5. IL-17F  6. IL-22 7. IL-1β 8. TNF-α 9. CCR6 |
| Nan, *et al* 2020 | C57BL/6 mice (female, N/A) | N/A | Psoriasis model (62.5 mg/d 5% IMQ, 10 d) | Cur-GA-silica, 22.78 mg/d, g.t.; 5% IMQ applied on the back, 62.5 mg/d, 7 d | CON group: purified water topically applied on the dorsal skin, 7 d IMQ group: 5% IMQ applied on the back, 62.5 mg/d, 7 d **Cur RDP** group: Cur raw drug powder applied on the dorsal skin 4 h after IMQ, 7 d | 1. PASI 2. Epidermal thickness 3. IL-17 |
| Badanthadka,  *et al* 2021 | C57BL/6, BALB/c, Swiss albino mice (female, 6/6) | 8–12 | Psoriasis model (62.5 mg/d 5% IMQ, 6d) | 1% Cur gel, 150 mg on the back, 12.5 mg on the right ear, g.t.; IMQ, 62.5 mg on the back and 5 mg on the right ear, 6 d | CON group: Vaseline, 62.5 mg on the back and 5 mg on the right ear, 6 d IMQ group: 5% IMQ, 62.5 mg on the back and 5 mg on the right ear, 6 d **Clob** group: 120 mg on the back and 10 mg on the right ear, 6 d | 1. PASI 2. Skin thickness 3. Spleen weight 4. Superoxide dismutase 5. Catalase 6. Glutathione |
| **Abbreviations:** i.g., intragastrically; p.o., per os; d, day; qd, once a day; qid, quarter in die; g.t., external use; E, Experimental group; C, Control group; HE, hematoxylin and eosin; ASC, apoptosis-associated speck-like protein containing a caspase recruitment domain; NLRP3, nucleotide-binding domain and leucine-rich repeat containing protein-3; IL, interleukin; TNF, tumor necrosis factor; CCR6, C-C chemokine receptor 6; IHC, immunohistochemistry; TAC-CUR, tacrolimus and curcumin co-loading; TAC, tacrolimus; CUR-NPs, curcumin-loaded nanoparticles; CUR-NPs-gel, curcumin-loaded nanoparticles incorporated in silk fibroin hydrogel; Cur, curcumin; PASI, Psoriasis Area Severity Index; HA-ES, curcumin-loaded hyaluronic acid-modified ethosomes; IMQ, imiquimod ointment; CP, clobetasol propionate cream; Cur-GA-silica, curcumin and glycyrrhizic acid-loaded Syloid® 244FP EU produced using smartPearls technology; Clob, clobetasol; NF-κB, nuclear factor-κB. | | | | | | |
