# Supplementary material for: Efficacy and safety of curcumin in psoriasis: preclinical and clinical evidence and possible mechanisms
Source: Front Pharmacol. 2022 Aug 29;13:903160. doi: 10.3389/fphar.2022.903160 (PMC9477188; doi:10.3389/fphar.2022.903160)
Supplement: Supplementary file 6 [file Table3.docx]

| **Table S3. Characteristics of the included preclinical studies *in vitro*** | | | | | | |
| --- | --- | --- | --- | --- | --- | --- |
| **Author, year** | **Cell type** | **Cell origin (species)** | **Model (method)** | **Cell tracking method** | **Outcomes** | **Pathway** |
| Wang, *et al* 2019 (1) | HaCaT | Human normal skin immortalized KC | 25 ng/mL VEGF | CCK-8 (HaCaT cell growth)  WB (KLF6, p21)  FCM (cell cycle, apoptosis of HaCaT) | Cur inhibits proliferation of HaCaT cells, promotes apoptosis stimulated by VEGF, and downregulates KLF6 and p21 proteins. | N/A |
| Zhang, *et al* 2020 | HaCaT | Human normal skin immortalized KC | 25 ng/mL VEGF | CCK-8 (HaCaT cell growth)  PCR (miR-203) | Cur inhibits the proliferation and miR-203 expression of HaCaT cells induced by VEGF. | N/A |
| Song, *et al* 2014 | HaCaT | Human normal skin immortalized KC | N/A | SABC (P53, Bax, Fas)  WB (P53, Bax, Fas, Bcl-2) | Cur inhibits Bcl-2 protein in HaCaT cells and promotes P53, Bax, and Fas protein expression. | N/A |
| Wang, *et al* 2019 (2) | HaCaT | Human umbilical cord blood, human normal skin immortalized KC | N/A | WB (VEGF, cyclin D1, Bcl-2) MTS (HaCaT cell growth)  FCM (cell cycle and apoptosis of HaCaT) | Cur inhibits cell proliferation; promotes apoptosis; and inhibits the expression of VEGF, cyclin D1, and Bcl-2 proteins in HaCaT cells. | N/A |
| Zhao, *et al* 2015 | HaCaT | Human normal skin immortalized KC | IL-22 | CCK-8 (HaCaT cell growth) PCR (cyclin D1, cyclin E)  WB (P-STAT3, cyclin D1, cyclin E) | Cur inhibits HaCaT cell proliferation with or without IL-22 treatment, inhibits the proliferation of HaCaT cells by arresting cells in the S phase, affects cyclin D1 and cyclin E protein expression through post-transcriptional modifications, and downregulates P-STAT3.  Cur抑制HaCaT细胞增殖，无论是否经IL-22处理，通过将细胞阻滞在S期来抑制HaCaT细胞的增殖，通过转录后修饰影响细胞周期蛋白D1和细胞周期蛋白E的表达，并下调P-STAT3。 | Erk/MAPK, JAK-STAT3 |
| Varma, *et al* 2017 | HaCaT | Human normal skin immortalized KC | IMQ | MTT (cell viability)  PCR (IL-17, TNF-α, IFN-γ, IL-6, FLG, iNV) ELISA (IL-17, TNF-α, IFN-γ, IL-6) | Cur (25 and 50 µM) inhibits the proliferation of HaCaT cells and increases the gene expression of iNV and filaggrin.  Cur downregulates IL-17, TNF-α, INF-γ, and IL-6.  Cur enhances the skin barrier function by upregulating iNV and FLG. | N/A |
| Cho, *et al* 2007 | HaCaT | Human normal skin immortalized KC | TNF-α | RT-PCR (IL-1β, IL-6, TNF-α, IL-8)  WB (IL-1β, IL-6, TNF-α, IL-8, cyclin E, p38, MAPK, JNK, ERK) | Cur inhibits the expression of TNF-α-induced IL-1β, IL-6, and TNF-α, but not IL-8, and inhibits the TNF-α-induced cyclin E expression.  Cur inhibits the activation of MAPKs (JNK, p38 MAPK and ERK) and NF-κB in TNF-α-treated HaCaT cells.  Cur抑制TNF-α诱导的IL-1β、IL-6和TNF-α的表达，但不抑制IL-8，并抑制TNF-α诱导的细胞周期蛋白E的表达。  Cur抑制TNF-α处理的HaCaT细胞中MAPKs（JNK、p38-MAPK和ERK）和NF-κB的激活。 | NF-κB  or MAPKs |
| Esposito, *et al* 2015 | HaCaT | Human normal skin immortalized KC | 1–10 μm NLD | Cellular viability  WB (cyclin D1)  Immunocytochemistry (p65 subunit) | NLDs CUR inhibit the activation of NF-κB and cyclin D1.  CUR抑制NF-κB和细胞周期蛋白D1的激活。 | NF-κB |
| Hung, *et al* 2015 | HaCaT | Human normal skin immortalized KC | 0, 0.2, 0.4, 0.6, 0.8, or 1 mg pSG5.HA.mZac1 | RT-PCR (p21, p16, cyclin D1)  WB (Zac1, cyclin D1, p21, p16)  FACS cell cycle) | Cur inhibits ZAC, downregulates cyclin D1, decelerates cell cycle progression in psoriatic KCs, and arrests cells at the G1 phase of the cell cycle.  Cur抑制ZAC，下调细胞周期蛋白D1，减缓银屑病KCs的细胞周期进程，并将细胞阻滞在细胞周期的G1期 | N/A |
| Yuyun, *et al* 2021 | HaCaT | Human normal skin immortalized KC | TNF-α | WB (p38, ERK, JNK)  MTT (proliferation of HaCaT cells)  ELISA (IL-6, IL-8, IL-1β) | MPA-CUR inhibits IL-6, IL-8, and IL-1β in TNF-α induced HaCaT cells.  MPA-CUR inhibits the suppression of p38, ERK, and JNK phosphorylation and hyperproliferation.  MPA-CUR 抑制 TNF-α 诱导的 HaCaT 细胞中的 IL-6、IL-8 和 IL-1β。  MPA-CUR 抑制 p38、ERK 和 JNK 磷酸化和过度增殖的抑制。 | MAPK |
| Sun, *et al* 2012 | HaCaT | Human normal skin immortalized KC | TNF-α | ELISA (IL-6, IL-8)  FC (TRAIL receptors)  WB (TRAIL) | Cur inhibits the TNF-α-induced production of IL-6/IL-8 in HaCaT cells.  Cur inhibits the expression of TNF-α-induced NF-κB dependent anti-apoptotic proteins and induces apoptosis in HaCaT cells.  Cur enhances TRAIL expression. | NF-κB |
| **Abbreviations:** KC, keratinocyte; WB, western blot; PCR, quantitative real-time polymerase chain reaction; ELISA, enzyme-linked immunosorbent assay; FCM, flow cytometry; iNV, involucrin; CCK-8, Cell Counting Kit-8; IL, interleukin; TNF, tumor necrosis factor; Zac1, zinc-finger protein that regulates cell cycle arrest and apoptosis 1; FLG, filaggrin; DLR, dual luciferase reporter assay; TRAIL, TNF-related apoptosis-inducing ligand; FACS, fluorescence-activated cell sorting; MAPK, mitogen-activated protein kinase; EMSA, electrophoretic mobility shift assay; FC, flow cytometric; SABC, streptavidin–biotin complex; KLF6, Kruppel-like factor 6; VEGF, vascular endothelial growth factor; NLDs CUR, nanostructured lipid dispersions containing curcumin; MPA-CUR, mycophenolic acid-esterified curcumin. | | | | | | |
